# The influence of co-encapsulated *L. plantarum* and *Silybum marianum* seed extract on the physicochemical properties of synbiotic cheese during ripening

**DOI:** 10.1016/j.fochx.2024.101674

**Published:** 2024-07-23

**Authors:** Maryam Bakhtiyari, Zohreh Hamidi-Esfahani, Mohsen Barzegar

**Affiliations:** Department of Food Science and Technology, Faculty of Agriculture, Tarbiat Modares University, Tehran, Iran

**Keywords:** Co-encapsulation, *L. plantarum*, *Silybum marianum* seed extract, Physicochemical properties, Synbiotic cheese

## Abstract

The effect of *Silybum marianum* seed extract (SMSE), added freely or in co-encapsulated with L. *plantarum* (MT, ZH593), on cell survivability, physicochemical and textural parameters in synbiotic cheeses for 60 days at 4 °C were studied. Incorporated cheeses with free, single encapsulated, and co-encapsulated probiotic + SMSE experimented a reduction of 3.19, 1.23, and 0.76 log CFU/mL for the cell survivability and their antioxidant activity reached 15.19, 16.26, and 31.73%, respectively, at the end of the storage. Decrease in hardness, cohesiveness, and springiness of the cheese containing free probiotic + SMSE upon compression during storage revealed proteolysis pattern and pH development being the most effective agents while whey percentage and moisture loss were the most effective agents in the rest of the cheeses. Overall, microcapsules containing L. *plantarum* and SMSE propose an easy and efficient delivery vehicle for the transition of bio-compounds into cheese as a novel synbiotic food.

## Highlights

• Extract had not significant effect on the physicochemical parameters of cheeses.
• Extract increased cell viability and antioxidant activity in synbiotic cheeses.
• Free *L. plantarum* showed high proteolysis activity and low viability in cheeses.
• Co-encapsulated *L. plantarum* showed low pH development and caused a harder curd.
• Cohesiveness values of cheeses indicated more elastic manner of cheeses.

## 1 Introduction

Applying plant extracts, polyphenol compounds, and antioxidants as new alternatives for non-digestible dietary ingredients known as prebiotics is recently the hot subject for investigation of scientific (Raddatz & de Menezes, 2021). *Silybum marianum* (milk thistle, Gaertn, Asteraceae) is one of the most widely distributed medicinal herbals in the pharmaceutical industry and also one of the top ten herbals applied in dietary supplements. The *Silybum marianum* seed extract (SM SE) known as silymarin, possesses various flavonoids and flavonolignans namely, silybinin, isosilybinin, sillychristin, silydianin, taxifolin, and quercetin with high antioxidant content and free radical scavenging properties which were used to treat several diseases such as liver, kidney, spleen, and gallbladder disorders (Mudge et al., 2015). Although, low bioavailability and chemical instability intensely confine its application in the food industry. One of the important common challenges between probiotic bacteria and polyphenol compounds are incorporation into fortified foods while retaining their bioactivity under adverse conditions (such as oxygen, humidity, temperature, low pH, and digestive enzymes), and during food processing as well as storage period. To overcome these troubles, the encapsulation method has been applied (Ribeiro et al., 2016). To achieve prosperous encapsulation, wall substances must be properly characterized as it will influence the morphology and stability of the microbeads during the storage period (Raddatz & de Menezes, 2021). The co-encapsulation of two or further bio-compounds in a univalent matrix causes increment bioactivity rather than the single encapsulation.

Cheese is one of the most efficient dairy carriers for the conservation of bio-compounds. Cheese texture is a main factor for consumer acceptance. Besides chemical composition, the organizational components of the cheese, particularly the protein matrix influence its texture (Ong et al., 2006). On the other hand, the incorporation of bio-compounds in cheese manufacturing is expected to affect its physicochemical and textural properties. The use of single encapsulated plant extracts and bioactive components in cheese products has previously been investigated (Ribeiro et al., 2016; Sharifi et al., 2021).

The use of the SMSE as a bio-compound in synbiotic products has not been already investigated. Because of the synergistic effect between L. *plantarum* (MT, ZH593) and SMSE (Bakhtiyari et al., 2022), it is suggested the application of these bio-compounds in the medicinal and synbiotic foods. Unexpectedly, no study illustrates the potential of cheese as a matrix carrier for *co*-encapsulated polyphenol compounds and probiotic bacteria, specifying it as a synbiotic food. Thus, for the first one in this study was successfully manufactured a novel whey less Feta-type cheese as a functional food incorporated with free, single encapsulated and co-encapsulated probiotic cells with SMSE, analyzing its potential as an ideal synbiotic food. For this purpose, survivability of probiotic cells, antioxidant activity, physicochemical composition alterations, proteolysis trend, and textural characteristics of cheeses during 60 days of storage period were evaluated. The morphological characteristic and stability of the whey protein isolate/chitosan coated alginate microbeads was also examined in model saline solution before adding into cheese samples for 30 days at 4 °C.

## 2 Materials and methods

### 2.1 Materials

Frozen native cultures of L. *plantarum* (MT, ZH593) were utilized from sample stocks that previously were isolated from Iranian koozeh traditional cheese (Tavakoli et al., 2017). Folin-Ciocalteau phenol reagent, sodium alginate, bile salt, gallic acid, and low molecular weight chitosan were purchased from Sigma-Aldrich Chemical Co. (St. Louis, MO, USA). De Man, Rogosa, and Sharpe broth media (MRS broth) and MRS agar, calcium chloride, calcium carbonate, and sodium chloride were provided by Merck Chemical Co. (Darmstadt, Germany)**.** Whey protein isolate (93% protein content, Davisco Inc., Le Sueur, USA)**.** All other chemical solvents used were of analytical grade.

### 2.2 Preparation of *Silybum marianum* seed extract (SMSE)

Dried *Silybum marianum* seeds were purchased from a local medicinal plant store in Alborz province, Iran. Polyphenol compounds of seeds after grounding and defatting in a soxhlet set (Behr, Germany) were extracted with methanol (80%) at 45 °C for 30 min in a sonicating bath (400 W, 20KHz, Iran). Extracted seeds after centrifugation (4472 *g*, 5 min at 4 °C) were evaporated with a rotary evaporator and freeze-dried for 24h (Mudge et al., 2015).

### 2.3 Co-encapsulation

Microbeads were fabricated using the internal/emulsification gelation procedure (Poncelet, 2001). 200 μL of calcium carbonate slurry (2%) and 200 μL of cell suspension (10.28 ± 0.38 log CFU/mL) were added into 10 mL of sodium alginate (1.38%) containing the SMSE (1996.3 μg/mL). The applied conditions such as concentrations of sodium alginate, SMSE, and calcium chloride were optimized and adapted from a similar study performed by Bakhtiyari et al. (2022).

Alginate solution was transmitted into 40 mL sunflower oil comprising 1% tween 80 while stirring (600 rpm). After a few minutes, 10 mL of sunflower oil comprising 35 μL glacial acetic acid was added to the emulsion. Then 80 mL of calcium chloride (82.74 mM) was gently added. After sieving (400 mesh) and rinsing with saline solution (0.85%), microbeads were coated with whey protein isolate (WPI) and chitosan (CS). For coating with CS, 0.5 g CS was dissolved in 90 mL acidified distilled water with 0.4 mL glacial acetic acid (0.1 mol/L). After adjusting pH to 5.7–6 using NaOH, the CS solution was autoclaved and filtered. Then 5 g of prepared microbeads was immersed in 100 mL of the CS solution while shaking (Heidolph, Germany) at 100 rpm, 37 °C for 40 min. After sieving and rinsing (0.85% saline), it was stored at 4 °C (Zhang et al., 2005).

To coat with WPI, prepared microbeads (0.2 g) were transmitted to 10 mL of the WPI solution (2.76%; pH 7.0, adjusted by NaOH) with gently shaken (Heidolph, Germany) for 15 min. After sieving and rinsing, it was stored in a 4 °C (Gbassi et al., 2009).

### 2.4 Cheese making

The cheeses were manufactured on a laboratory scale according to the method of Lashkari et al. (2020) with a few modifications. Fresh cow milk, and cream were obtained from Taravat Dairy Co. (Pakdasht, Iran) and milk protein concentrate (MPC) was obtained from Pegah Dairy Co. (Tehran, Iran). Cream, MPC, WPC, and NaCL were mixed with ratios of 45.6, 11.7, 2.7, and 1.5%, respectively, with fresh milk at 38.5%. After heating at 45 °C for 1 h, the mixtures were pasteurized at 72 °C for 15 s. After cooling to 35 °C, starter culture (LBB, Hansen, Denmark) and CaCl_2_ (0.01 and 0.2 g / kg of milk, respectively) were transferred into the milk. Then it was held for 30 min at 35 °C and the coagulation was done via rennet (0.04g / L of milk; Enzy-Max, 3400 IMCU/g, Chr. Hansen, Denmark). Afterward, each container was sealed by aluminum foil hermetically. Sealed cups were placed at 40 °C for 4 h and then stored at 4 °C. Cheese samples were taken for analyses at time intervals of 1, 10, 20, 30, 40, 50, and 60 days.

#### 2.4.1 Functionalization of whey-less feta cheeses with bio compounds

Four types of whey-less Feta cheese were processed in triplicate: (1; C) control with the addition of only commercial starter, (2; FreeLPE) synbiotic cheeses containing free L. *plantarum* and SMSE, (3; EnLP) probiotic cheeses containing single encapsulated L. *plantarum,* (4; Co-enLPE) synbiotic cheeses containing co-encapsulated *L. plantarum* together with SMSE. Free, single encapsulated and co-encapsulated *L. plantarum* together with SMSE powder (1%) was incorporated into the milk simultaneously with inoculation of the starter, for the production of samples Free LPE, EnLP and Co-en LPE. The number of L. *plantarum* cells in these type of cheese was about 3 × 10^9^ CFU /mL. In the following, the process was carried out according to the mentioned method.

### 2.5 Analyses

2.5.1. Characterization of beads

The morphologies and average diameter of the 150 randomly selected alginate microbeads and WPI/CS coated microbeads were measured using an optical microscope (Nikon, Tokyo, Japan) connected with a digital camera (Olympus Optical, Tokyo) (Shi et al., 2013).

#### 2.5.1 Storage stability of the free, single encapsulated and co-encapsulated L. *plantarum*

The survival of free probiotic bacteria, alginate microbeads, WPI and CS coated microbeads containing cells with or without SMSE, was tested by incubating 5 mL of free***,*** encapsulated and co-encapsulated cells dispersed in a 45 mL of sterile saline solution (0.5 g/100 mL) and kept at 4̊ C. 1 mL of sample was taken out to count the total number of viable cells during storage every 5-days for 30 days (Vodnar & Socaciu, 2014).

#### 2.5.2 Survival of free, encapsulated, and co-encapsulated *L. plantarum* in whey-less Feta cheese during storage

To count the survival *of* L. *plantarum* in Feta cheeses during 60 days, every 10 days 5 g of the cheese samples was put into a stomacher bag comprising 45 mL of a 2% trisodium citrate solution and homogenized with a stomacher (Seward Laboratory, London, UK) at 260 rpm for 9 min. Serial dilutions were decimally done in saline solution and samples were pour-plated in MRS agar (comprising 0.15% bile salt to hinder the growth of starter in cheese) and incubated at 37 °C for 48 h (Motalebi Moghanjougi et al., 2020).

#### 2.5.3 Evaluation of the total phenolic content (TPC) and antioxidant activity (AA) of the cheeses during storage

To evaluate the phenolic content of the Iranian cheeses during 60 days, every 10 days 10 g of the cheeses was extracted with 50 mL of methanol (95%) using a homogenizer (IKA Co., Germany) with a speed of 10,000 rpm for 1 min. After centrifugation (3622*g*, 10 min) of homogenized samples (Rashidinejad et al., 2013), the extract was applied for evaluation of TPC using the Folin-Ciocalteu reagent, by a Cary 60 UV/Vis spectrophotometer (Agilent Technologies, USA). The complete method has already been explained by the authors (Bakhtiyari et al., 2022).

The antioxidant activity of the extract was evaluated using DPPH based on the method Brand-Williams and others (Brand-Williams et al., 1995). 0.1 mL of extract was mixed with 3.9 mL of 0.1 mM DPPH. After 30 min absorbance of extract was analyzed at λ = 517 nm with a Cary 60 UV/Vis spectrophotometer (Agilent Technologies, USA).

#### 2.5.4 Chemical and biochemical properties analysis

Cheese samples were evaluated in triplicate during the storage period for fat using the Gerber method, and total protein content by the Kjeldahl method. Moisture content was obtained using oven-drying (102 °C) up to fixed weight. The pH was measured by a pH meter (Metrohm, 827). The syneresis percentage was measured by dividing the weight of the expelled liquid by the total weight. The titrable acidity of cheeses was obtained by the Iranian National Standard (2852) and represented as a percentage of lactic acid (AOAC International, 2010).

According to Desmazeaud et al. (1976), the progress of proteolysis of the cheese samples throughout storage time was studied by evaluating the amount of soluble nitrogen (SN) fractions, such as SN at pH 4.6 (pH 4.6-SN) and SN in trichloroacetic acid (TCA-SN). In each fraction, the total nitrogen (TN) was measured by the Kjeldahl.

For fractionation at pH 4.6, 40 mL of a sodium citrate solution (0.5 M) was added to the 10 g of cheese and was held for 30 min at 40 °C, then was adjusted to pH = 4.6 by hydrochloric acid (1 N). The suspension was centrifuged (3622*g*, 10 min) and filtered by Whatman No. 40 paper. Finally, nitrogen soluble was analyzed by the Kjeldahl method.

For fractionation in trichloroacetic acid (TCA-SN; Non-protein nitrogen), 4 mL of a solution of TCA (60%) was added to 16 mL of the filtrate obtained from the SN-pH 4.6. Then the mixture was kept for 1 h at room temperature and was centrifuged as described above.

#### 2.5.5 Instrumental texture profile analysis (TPA)

The texture parameters of cheeses were measured using a texture analyzer (CT V1.5; Brookfield Engineering Labs), with a load cell and trigger load of 10 kg and 0.50 N, respectively. Block samples (30 mm height and 20 mm diameter) were compressed to 30% of their initial height at a rate of 1 mm/s by two-bite compressions using a cylindrical probe (TA5) of 12.7 mm^2^ area. The pre-test speed, test speed, and post-test speed were 2, 1, and 1 mm/s, respectively. The measured parameters consisted of cohesiveness, hardness, adhesiveness, springiness, gumminess, and chewiness, which were acquired with the Texture Expert for Windows software version 1.20 (Stable Micro Systems**).**

### 2.6 Statistical analyses

The results are given as the mean ± standard deviation of triplicate trials. To compare the data, analysis of variance (ANOVA) was conducted using SPSS 18. The comparison between the means was carried out by Duncan's multiple range test at a confidence level of *p* < 0.05.

## 3 Results and discussions

### 3.1 Morphological analysis of the microbeads

**Fig. 1:**
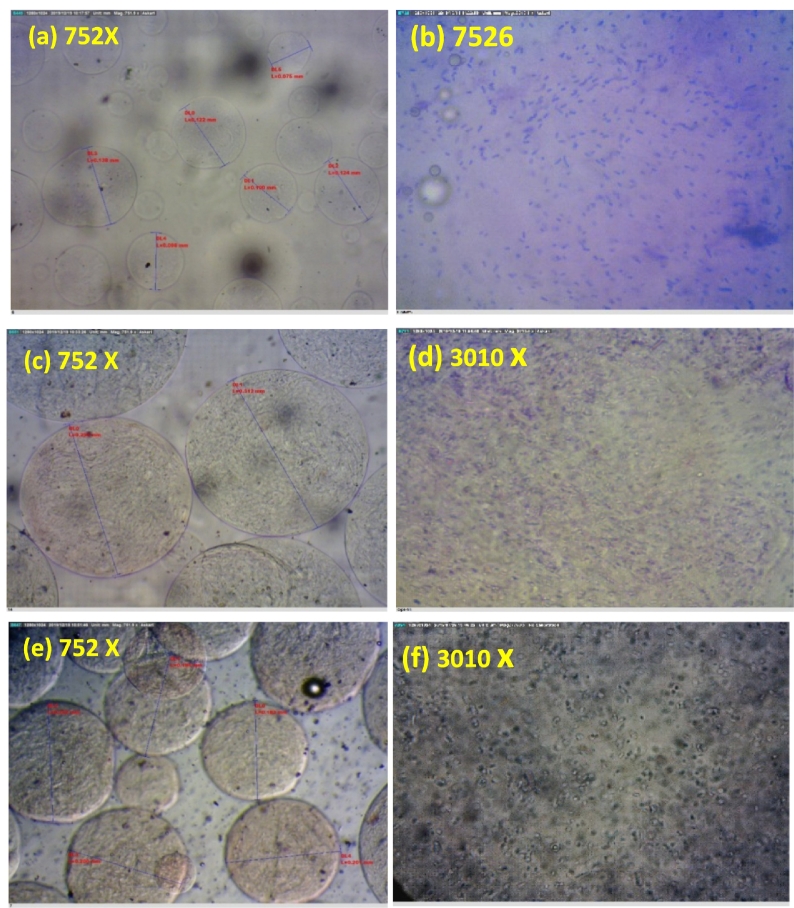
Optical microscopic images of alginate microbeads, WPI and CS coated microbeads (a,c and e) at 752 × magnification. (b) 7526, (d) 3010 and (f) 3010 × magnification distribution of L. *plantarum* (MT, ZH593) embedded in the microbeads.

Fig. 1 shows the shape and distribution size of the alginate microbeads, WPI, and CS-coated microbeads. Image of microbeads at 752 × magnification showed that the microbeads had a regular globular structure and maintained their shape after extra covering. Alginate microbeads, WPI, and CS-coated microbeads had size ranges of 144.29 ± 16.8, 283.46 ± 25.7, and 211.95 ± 31.6 μm, respectively. Image of microbeads at 7526 and 3010× magnification showed that the microbeads loaded with rod-shaped cells uniformly inserted in the microbeads. The significant increase in the size of coated microbeads indicates the success of the coating process and the formation of ionic interactions. The size of wet coated microbeads ranged from 181 to 308 μm, representing good potential for utilization in food systems as supplements. However, particle size above 100 μm is favorable in order to utilization in food product to maintain their sensory and textural characteristics. The diameter of microbeads ranging from 250 μm to 1 mm could result in sandiness in yogurt with liquid food matrices (Shi et al., 2013). Although larger microbeads can be applied in solid food matrices such as cheese and chocolate.

### 3.2 Storage stability of the free, encapsulated and co-encapsulated L. *plantarum*

**Fig. 2:**
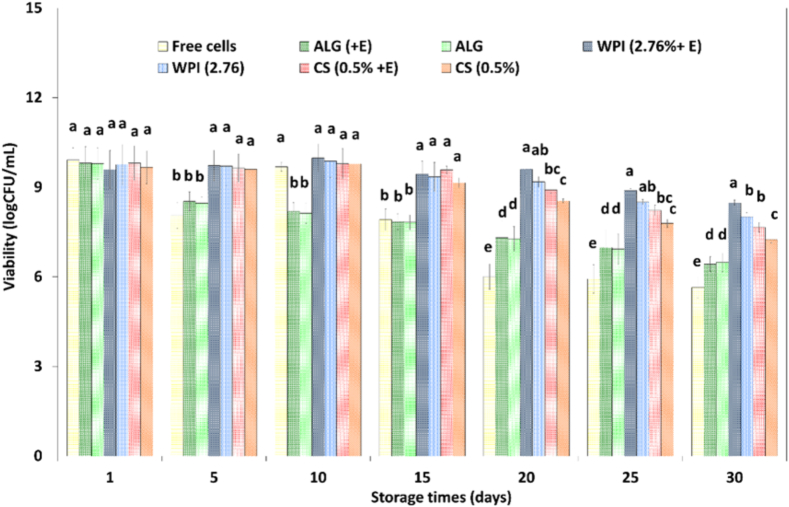
Storage stability (log CFU/mL) of free *L. plantarum* (MT, ZH593), alginate microbeads and WPI/CS coated microbeads containing cells with or without SMSE in sterile saline solution (0.5 g/100 mL) during storage for 30 days at 4 ° C. ALG, Alginate microbeads without coating containing only L. *plantarum*; WPI, Alginate microbeads coated with whey protein isolate (2.76 g/100 mL) containing only *L. plantarum*; CS, Alginate beads coated with chitosan (0.5 g/100 mL) containing only L. *plantarum*. (+E) in treatments means together with *silybum marianum* seed extract. ^⁎⁎^ Means with different letter above each column are significantly different (P ≤ 0.05). The values represent the means of three samples (n = 3).

The role of SMSE and encapsulating coatings on retaining survival cells during storage at 4 °C has been studied (Fig. 2). Probiotic bacteria cells before being applied in functional food products may be stored for a long time. There are no prior researches on the possibility of using SMSE for enhancing probiotic stability during refrigerated storage. According to Fig. 2, the survival of free and alginate encapsulated L. *plantarum* cells decreased sharply from 9.91 ± 0.41 to 5.64 ± 0.36 log CFU/mL (about 4.3 log unit reduction) and from 9.79 ± 0.53 to 6.48 ± 0.31 log CFU/mL (about 3.4 log unit reduction) after 30 days' storage at 4 °C, respectively. Microbeads coating with WPI and CS significantly enhanced the viability of cells as the viable bacteria were reduced from 9.76 ± 0.67 to 8.01 ± 0.14 log CFU/mL and from 9.67 ± 0.55 to 7.25 ± 0.11 log CFU/mL, respectively. For L. *plantarum* survivability at 4 °C, chitosan as not as effective as whey protein isolate as encapsulating coating, while significant enhancement was obtained toward the alginate microbeads. This may be attributed to production of the smaller CS microbeads relative to the WPI structures (Fig. 1). Due to the formation of a denser hydrogel network, microbead coatings reduced the speed of diffusion and caused a more retarded release of cells from the microbeads. Shi et al. (2013) reported that with increment microbeads diameter enhanced protective effect versus environmental agents, while, oversized microbeads lead to unsuitable mouthfeel. Other study reported that the large microbeads composed from different concentration of WPC and pectin provide high cell loading capacity and better protection for Bifidobacterium in harsh environment (Yasmin et al., 2019). Thickness of the coating layer in the alginate-gelatin microbeads was important for improving the protection of the *Lactobacillus salivarious* to environmental factors (Yao et al., 2017). Another agent is the oxygen permeability of the encapsulating coatings. Probiotics have anaerobic or microaerophilic metabolism and sensitive to the presence of oxygen, thus differences in oxygen permeability of the carbohydrate-based and protein-based matrixes may lead to the obtained results. Similar results were reported by López-Rubio et al. (2012) who that indicated greater viability of encapsulated *Bifidobacterium animalis* using WPC as the encapsulating matrix comparing with pullulan and demonstrated higher protection ability of WPC than pullulan at two storage temperatures 4 °C and 20 °C. After sampling and opening of the microbeads vessel may have facilitated the exposure to oxygen. It is clear that because of the exposure to air and the liquid medium, the bigger WPI microbeads was able to further delay oxygen penetration to encapsulated probiotic relative to the CS. It is also reported that the WPI (2.76%) coating importantly provided higher protection for core (*L. plantarum* and SMSE) relative to CS (0.5%) in simulated gastrointestinal fluids (Bakhtiyari et al., 2022).

No significant difference (*P* ≤ 0.05) was observed in the cell population in the CS and WPI coated microbeads with or without SMSE before of the days of 15 and 20 of storage period, respectively, while afterward significant difference was observed. WPI and CS coated microbeads containing cells and SMSE experimented the lowest loss of viability 1.12 log CFU/mL and 2.15 log CFU/mL, respectively, at end of the storage. The cell loss was obtained about 1.75 and 2.42 log for WPI and CS coated beads (SMSE-free) at end of the storage. According to these results, the addition of SMSE increased the survival of the encapsulated L. *plantarum* for coated beads (about 0.37–0.46 log cycles) during storage at last times (after of days of 15) of storage period. The different chemical compositions of SMSE are related to these findings. SMSE is a main source of silymarin which is a complex of the 8 types of flavonolignans. In an already study, free SMSE showed high antioxidant activity of 404.9 ± 15.4 μg/mL (on-based IC50) and the high content of phenolic compound (361.4 ± 7.8 mg gallic acid equivalent /10 g of seed). Also, co-encapsulated SMSE showed a high antioxidant activity of 51.92 ± 2.14% (Bakhtiyari et al., 2022). No improvement effect of SMSE in alginate beads was shown. Equal viability for alginate beads (with or without extract) could be related to a fast release of the extract (up to 51%) after 15 days in comparison to a slow release of the extract (11.39 ± 0.74 and 20.47 ± 2.12%) from WPI and CS coated beads at end of the storage (data are not shown). For this purpose, the stability of the *co*-encapsulated polyphenol compounds together with L. *plantarum* in alginate microbeads and WPI/CS coated microbeads during 30 days of storage at 4 °C was assessed by the Folin-Ciocalteau procedure (data are not shown). According to the results obtained, a high release rate of the extract resulted in the same cell survival even with addition of SMSE (Bakhtiyari et al., 2022). The probiotic strains, concentration and chemical structure of polyphenols are the most effective factors on probiotic growth by polyphenols. According to the results that were previously published, at 1996.3 μg/mL concentration of the SMSE (optimum condition) causes to production of spherical beads with the highest cell viability (about 79.84%) and the highest AA (Bakhtiyari et al., 2022). A few studies reported an important increase in probiotic growth with the adding of phenolic extract during refrigerated storage (Chaikham, 2015; Shinde et al., 2014; Vodnar & Socaciu, 2014). Phenolic extract, as an antiradical agent moderates oxidative stress caused by cellular metabolic activities. However, a detrimental effect of pure quercetin (standard), inhibiting *L.gasseri* and *B.bifidum* in ALG-CS capsules was reported by Chávarri et al., 2010. Therefore, not all polyphenol extracts and subcategories excite the viability of the probiotic population.

### 3.3 Survival of free, encapsulated, and co-encapsulated L. *plantarum* in cheese samples during storage at 4 °C

**Fig. 3:**
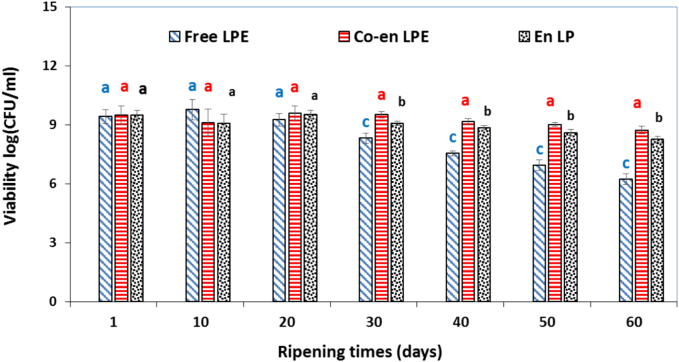
Survival of free, single encapsulated and co-encapsulated *L. plantarum* with SMSE in cheese samples during storage at 4 °C. The values represent the means of three samples (*n* = 3). Free LPE, containing starter culture along with free *L. plantarum* (MT, ZH593) and SMSE; Co-en LPE, containing starter culture along with co-encapsulated L. *plantarum* (MT, ZH593) and SMSE; En LP, containing starter culture along with single-encapsulated *L. plantarum* (MT, ZH593).

Comparing with CS, the higher cell viability (Fig. 2) and lower SMSE release of the WPI coated microbeads (11.39 ± 0.74%; data are not shown) at end of the storage time in both the sailin solution and in the simulated gastrointestinal fluids (Bakhtiyari et al., 2022) obviously showed the high protective effect of the studied protein matrix. Therefore, WPI coated microbeads was selected to functionalize whey-less Feta cheeses. There is a lack of researches studding the effect of SMSE (free, encapsulated or co-encapsulated) on viability of probiotic population in food products (especially cheese). Also, other phenolic compounds along with probiotics (encapsulated or co-encapsulated) in the different cheese has not been studied. Survivor of the free, single encapsulated, and co-encapsulated *L. plantarum* with SMSE were evaluated after aseptically transferring into whey-less Feta cheese following refrigerated storage for 60 days (Fig. 3). The viability of Free LPE on day 10 of ripening was observed to be higher than on day 0, also higher viability of Co-enLPE and EnLP on day 20 of ripening than on day 10 was observed. This irregular increase in the number of viable cells in the early days of ripening is probably due to lactose fermentation and acid production by starter bacteria, resulting in better growth of LAB probiotics under acidic conditions. It was reported that the initial increase in the growth of L. *acidophilus* in probiotic and C cheeses during the first 7 days of ripening might be due to the fermentation of lactose by starter lactococci. It is well known that lactobacilli grow best under acidic conditions (Kasimoǧlu et al., 2004). According to Fig. 3, the viability of free L. *plantarum* was decreased continuously from 9.41 ± 0.35 to 6.23 ± 0.26 log CFU/mL cells in the sample FreeLPE (a reduction of approximately 38%; *P* ≤ 0.05) after 60 days. The population of microencapsulated cells alone or together with SMSE reached 8.27 ± 0.15 and 8.73 ± 0.19 log CFU/mL in the samples EnLP and Co-enLPE, respectively, at the end of the storage. The survival of L. *plantarum* cells showed a reduction of 3.19, 1.23, and 0.76 log CFU/mL for free, single encapsulated, and co-encapsulated cells with SMSE in samples of FreeLPE, EnLP, and Co-enLPE, respectively, at the end of the storage. Different agents influence the stability of probiotic cells in cheese including the competitive effect of starter culture, low pH, presence of salt (sodium chloride), presence of oxygen, storage time, antagonistic effects of cheese flora and low temperature (Sharifi et al., 2021). The important challenge in the simultaneous inoculation of starter bacteria and nonstarter LAB in cheese is their sensitivity to the produced bacteriophages and the competition between starter and nonstarter LAB in providing nutritional resources and energy (Kasimoǧlu et al., 2004). The results of this study demonstrated that the loaded cheese with co-encapsulated, single encapsulated, and free cells had a lower decrease in the number of L. *plantarum* (0.00, 0.44, and 1.1 cycle log reduction) in the first month than the second month of storage (0.78, 0.80 and 2.08 cycle log reduction). According to these results, the addition of co-encapsulated SMSE together with cells increased the survival by about 0.36–0.45 log cycles at days 30, 40, 50, and 60 of the storage period while no improvement effect was observed during the early times of storage. Almost high cell survival in the last days of the storage period might be due to the antioxidant properties of SMSE in incorporated cheese with co-encapsulated bio-compounds. Santivarangkna et al. (2008) reported that lipid oxidation of the cell membrane tends to occur during the last storage course. On the other hand, oxidative and hydrolytic degradations may happen in the lipids of cheeses during storage. Farbod et al. (2013) stated that the thiobarbituric acid value of Iranian feta type cheese increased (up to 15%) during the 60 days of ripening at 5̊ C. High cell survival in cheese CoenLPE could be due to antioxidant properties of SMSE preventing lipid oxidation of cheese. The antioxidant activity of SMSE was attributed mainly to silybin, one of the major constituents of its silymarin composition. It was reported that the adding of the lavender flower powder in Gouda-type cheese decreased thiobarbituric acid (secondary lipid oxidation products) during ripening compare to control (Semeniuc et al., 2022). Moisture content, bacterial lipases and high level of fat in the studied cheese (42%; based on dry weight) could stimulate the generation of free fatty acids. In addition, the intense mechanical stresses such as the agitation actions resulted in oxygen entry in the final cheese. Also, homogenization of fat droplets distributes the fat, catalysts and oxygen among the lately regulated fat droplets, thus increases oxidation of lipid. On the other hand, thermal processing cleaves lipid peroxides to yield oxidation products. However, this study did not investigate the changes in oxidative stability and lipolysis of the cheeses during storage.

In an already study, the authors indicated that the addition of the SMSE increased about 0.7 log CFU/mL viability of co-encapsulated L. *plantarum* in simulated gastrointestinal fluids. A moderate increase of about 0.36–0.45 log CFU/mL obtained in synbiotic cheese could be due to a compact network of the texture, good buffering valency, and higher fat and protein content of cheese which may propose a conservation for the probiotic (de Oliveira et al., 2012) relative to simulated gastrointestinal fluids. Other reason could be due to prebiotic source of SMSE to maintaining probiotic viability. The ability of the LAB (*L. plantarum* in particular) to glycosylate (degradation or depolymerization) phenolic compounds to phenolic acids, flavonoid glycosides and tannin which are biologically more active has been previously reported (Ricci et al., 2019). On the other hand, physicochemical specifications of SMSE (multiple hydroxyl groups) could postpone the diffusion of L. *plantarum*, through the creation of interactions by hydrogen and covalent bonds with the microbeads wall (alginate and WPI), reinforcing microbeads structure. The next explanation could be due antimicrobial effect of the SMSE for reduction of the competitive microorganisms in cheese during storage. Many studies have reported that the SMSE showed an inhibition effect against *Escherichia coli, Pseudomonas aeruginosa, Staphylococcus aureus*, molds, yeast and other pathogenic bacteria in food industry (Abdelazim, 2017). Furthermore, were reported that the significant inhibitory effect of the studied L. *plantarum* MT.ZH593 on the growth of the tested foodborne pathogens including *Escherichia coli PTCC5052, Salmonella enterica, Enetrococcus hirea, Staphylococcus aureus* and *Pseudomonas aeruginosa*, which be attributed to the production of bacteriocins and organic acids, showing good activity in acidic media (Tavakoli et al., 2017). The synergistic effects were expected between SMSE and L. *plantarum* regarding to their antimicrobial activity in CoenLPE cheese during storage.

### 3.4 Evaluation of the total phenolic content (TPC) and antioxidant activity (AA) of the cheeses during storage at 4 °C

The TPC and AA of Feta-type cheeses are indicated in Table 1. A significant effect of the SMSE adding to the milk (*p* < 0.05), encapsulation type (p < 0.05), and aging time (p < 0.05) was found for cheeses in terms of the TPC and AA. All of the cheeses showed a significant increase in the TPC and AA during the ripening time, which had a good match with secondary proteolysis (TCA-SN indicator; Table 2) with the exception of the cheese FreeLPE with a significant decrease at 20 days. With increasing the AA the viability of probiotics in cheeses was significantly reduced during ripening (*P* < 0.05) shown in Fig. 3. It could be inferred that functional properties and the number of L. *planrarum* have little effect on the AA of the cheese sample during ripening. This increase could be due to casein degradation by extracellular protease and intracellular peptidase produced by the L. *planrarum*, thus conferring peptides with antioxidant capacity.

**Table 1:** Evaluation of the total phenolic content (TPC) and antioxidant activity (AA; by DPPH assay) of the cheese samples during storage at 4 °C.

	Time of storage ^†^_‡_ (days)⁎						
	1	10	20	30	40	50	60
*TPC:*							
C⁎	53.03_F_^d^ ± 0.82	65.01_E_^c^ ± 2.99	58.41_F_^d^ ± 1.62	78.16_D_^c^ ± 2.21	96.19_C_^d^ ± 4.81	131.70 _A_^d^ ± 5.09	126.25_B_^d^ ± 1.02
Free LPE	284.27_B_^a^ ± 1.12	282.34_B_^a^ ± 21.70	214.58_C_^b^ ± 3.14	228.34_C_^b^ ± 2.75	302.12_B_^b^ ± 17.28	336.56_A_^b^ ± 6.58	327.51_A_^b^ ± 9.17
Co-en LPE	268.27_F_^b^ ± 2.09	294.93_E_^a^ ± 3.13	306.17_E_^a^ ± 4.09	325.28_D_^a^ ± 19.11	392.32_C_^a^ ± 5.59	416.34_B_^a^ ± 13.47	483.54_A_^a^ ± 11.76
En LP	71.62_F_^c^ ± 3.36	87.65_DE_^b^ ± 2.79	81.33 _E_^c^ ± 4.11	93.16 _D_^c^ ± 3.12	165.42 _C_^c^ ± 5.11	176.26 _B_^c^ ± 4.17	209.18_A_^c^ ± 3.21
*AA:*							
C	4.83_D_^c^ ± 0.59	6.28 _C_^b^ ± 0.76	6.90 _C_^d^ ± 0.23	8.15 _B_^c^ ± 0.74	8.49_AB_^c^ ± 0.58	9.62_A_^d^ ± 0.64	9.24_AB_^c^ ± 0.83
Free LPE	20.47 _A_^a^ ± 0.96	19.54 ^a^_A_ ± 0.82	12.39_C_^b^ ± 0.36	12.02^b^_C_ ± 1.38	14.28_B_^b^ ± 0.92	14.57^c^_B_ ± 0.51	15.19_B_^b^ ± 0.94
Co-en LPE	20.73^a^_D_ ± 1.15	20.06_D_^a^ ± 1.44	21.16^a^_D_ ± 0.27	24.38_C_^a^ ± 0.42	28.73^a^_B_ ± 1.46	27.56_B_^a^ ± 0.48	31.73^a^_A_ ± 1.07
En LP	6.78_E_^b^ ± 0.39	8.14^b^_DE_ ± 1.05	9.23_D_^c^ ± 0.82	10.95^b^_C_ ± 1.19	14.09_B_^b^ ± 0.78	15.75^b^_A_ ± 0.24	16.26_A_^b^ ± 1.29

† a, b, c–For each day of storage, different lowercase superscripts in a column signify significant differences (*P* ≤ 0.05) between different treatments.

‡ A, B, C–For each treatment, different capital superscripts in a row signify significant differences (*P* ≤ 0.05) between different days of storage.

⁎ C, control, containing only starter culture; Free LPE, containing starter culture along with free L. *plantarum* and SMSE; Co-en LPE, containing starter culture along with co-encapsulated L. *plantarum* (MT, ZH593) and SMSE; En LP containing starter culture along with encapsulated L. *plantarum* (MT, ZH593). TPC values are expressed as mg gallic acid equivalents (GAE) per 100 g of cheese, and antioxidant activity values are expressed as percentage (%).

**Table 2:** Means ± SD of The chemical composition of the enriched cheeses with free, single encapsulated and co-encapsulated cells and SMSE during storage for 60 days at 4̊ C.

	**Cheese samples**	**Storage times**^**†**^_**‡**_**(days)**⁎						
		**1**	10	20	30	**40**	**50**	**60**
**Moisture (%)**	C	63.13_A_^a^ ± 1.51	63.47_A_^a^ ± 1.27	62.07_B_^a^ ± 0.52	61.14_C_^a^ ± 1.24	62.63_AB_^ab^ ± 0.84	59.14_D_^ab^ ± 0.35	58.71_D_^ab^ ± 0.89
	Free LPE	65.16_A_^a^ ± 0.92	63.33_B_^a^ ± 0.62	62.31_BC_^a^ ± 0.72	61.42_C_^a^ ± 0.85	63.45_B_^a^ ± 0.72	60.75_D_^a^ ± 1.15	59.62_D_^a^ ± 0.36
	Co-en LPE	62.34_A_^a^ ± 0.65	62.57_A_^ab^ ± 0.54	61.89_AB_^a^ ± 0.79	61.28_B_^a^ ± 0.86	63.26_A_^a^ ± 0.76	60.15_B_^a^ ± 1.05	58.82_C_^ab^ ± 0.67
	En LP	63.09_A_^a^ ± 0.92	62.46_AB_^ab^ ± 1.25	61.68_BC_^a^ ± 1.24	62.08_B_^a^ ± 0.88	62.57_AB_^ab^ ± 0.46	59.39_C_^ab^ ± 0.54	58.95_C_^ab^ ± 0.44
**Synersis (%)**	C	2.36_CD_^b^ ± 0.25	0.52_E_^b^ ± 0.16	1.38_DE_^c^ ± 0.96	3.81_BC_^c^ ± 1.08	5.29_AB_^bc^ ± 1.12	4.80_AB_^b^ ± 1.24	6.02_A_^c^ ± 0.35
	Free LPE	3.85_D_^a^ ± 1.31	2.94_D_^a^ ± 1.24	5.46_C_^a^ ± 0.38	8.77_B_^a^ ± 0.46	7.95_B_^a^ ± 0.62	9.34_B_^a^ ± 0.32	10.77_A_^a^ ± 0.12
	Co-en LPE	1.65_E_^b^ ± 0.48	1.38_E_^ab^ ± 0.89	4.26_D_^b^ ± 0.39	5.75_BC_^b^ ± 1.13	4.81_CD_^c^ ± 0.51	6.52_AB_^b^ ± 1.27	7.33_A_^b^ ± 0.26
	En LP	1.72_C_^b^ ± 0.65	1.85_C_^ab^ ± 0.57	4.59_B_^ab^ ± 0.31	5.94_B_^b^ ± 0.22	6.20_AB_^b^ ± 0.32	5.78_B_^b^ ± 2.03	7.65_A_^b^ ± 0.81
**pH**	**C**	5.33_A_^a^ ± 0.09	5.11 _B_^a^ ± 0.05	4.98 _C_^a^ ± 0.11	4.61 _D_^a^ ± 0.06	4.27 _F_^a^ ± 0.07	4.43_E_^a^ ± 0.04	4.51 _DE_^a^ ± 0.05
	Free LPE	5.13_A_^a^ ± 0.10	4.89 _B_^a^ ± 0.12	4.72 _C_^a^ ± 0.07	3.94 _D_^c^ ± 0.09	3.71 _E_^c^ ± 0.11	3.75_E_^c^ ± 0.06	3.70 _E_^c^ ± 0.03
	Co-en LPE	5.27_A_^a^ ± 0.06	4.94 _B_^a^ ± 0.10	4.91 _B_^a^ ± 0.08	4.32 _C_^b^ ± 0.07	4.02 _E_^b^ ± 0.10	4.14_DE_^b^ ± 0.05	4.27_CD_^b^ ± 0.08
	En LP	5.29_A_^a^ ± 0.11	4.96 _B_^a^ ± 0.14	4.93 _B_^a^ ± 0.13	4.36 _C_^b^ ± 0.06	4.07 _E_^b^ ± 0.08	4.18_DE_^b^ ± 0.03	4.25_CD_^b^ ± 0.04
**Acidity**	**C**	0.43_F_^a^ ± 0.06	0.53 _E_^a^ ± 0.05	0.68 _D_^b^ ± 0.04	1.04 _B_^c^ ± 0.03	1.26 _A_^c^ ± 0.09	1.07_B_^c^ ± 0.04	0.84_C_^c^ ± 0.05
	Free LPE	0.49 _F_^a^ ± 0.04	0.59 _E_^a^ ± 0.03	0.82 _D_^a^ ± 0.01	1.40 _C_^a^ ± 0.03	1.61 _A_^a^ ± 0.07	1.50_B_^a^ ± 0.03	1.58_A_^a^ ± 0.07
	Co-en LPE	0.46_F_^a^ ± 0.02	0.58 _E_^a^ ± 0.05	0.70 _D_^b^ ± 0.08	1.23 _B_^b^ ± 0.03	1.40 _A_^b^ ± 0.08	1.29_B_^b^ ± 0.02	1.08_C_^b^ ± 0.06
	En LP	0.47_E_^a^ ± 0.07	0.54_E_^a^ ± 0.08	0.73 _D_^ab^ ± 0.04	1.22 _B_^b^ ± 0.08	1.42 _A_^b^ ± 0.06	1.25_B_^b^ ± 0.04	1.05_C_^b^ ± 0.03
**Fat (%)**	**C**	15.24_BC_^a^ ± 0.57	15.09_C_^a^ ± 0.42	14.34_D_^b^ ± 0.31	15.46_BC_^a^ ± 0.44	16.32_A_^a^ ± 0.31	16.25_A_^a^ ± 0.23	15.84_AB_^a^ ± 0.42
	Free LPE	15.74_CD_^a^ ± 0.38	16.24_AB_^a^ ± 0.41	16.65_A_^a^ ± 0.24	15.42_D_^a^ ± 0.16	15.95_BCD_^a^ ± 0.31	16.14_AB_^a^ ± 0.13	16.34_AB_^a^ ± 0.33
	Co-en LPE	15.14_A_^a^ ± 0.24	15.49_A_^a^ ± 0.27	14.86_A_^b^ ± 0.33	14.94_A_^a^ ± 0.31	15.36_A_^a^ ± 0.46	15.67_A_^a^ ± 0.32	15.53_A_^a^ ± 0.51
	En LP	15.35_D_^a^ ± 0.29	15.50_CD_^a^ ± 0.50	15.91_BCD_^a^ ± 0.54	15.96_BCD_^a^ ± 0.23	16.28_AB_^a^ ± 0.61	16.49_A_^a^ ± 0.43	16.68_A_^a^ ± 0.59
**Protein (%)**	C	11.53_A_^a^ ± 0.16	11.40_A_^a^ ± 0.47	11.21_A_^a^ ± 0.32	11.14_A_^a^ ± 0.57	10.93_A_^a^ ± 0.48	10.71_A_^a^ ± 0.30	10.64_A_^a^ ± 0.32
	Free LPE	11.73_A_^a^ ± 0.28	11.64_A_^a^ ± 0.36	11.42_AB_^a^ ± 0.15	11.34_AB_^a^ ± 0.43	11.31_AB_^a^ ± 0.44	10.97_B_^a^ ± 0.35	10.82_B_^a^ ± 0.18
	Co-en LPE	11.66_A_^a^ ± 0.31	11.62_A_^a^ ± 0.17	11.54_AB_^a^ ± 0.23	11.29_AB_^a^ ± 0.39	11.05_BC_^a^ ± 0.26	10.64_C_^a^ ± 0.32	10.55_C_^a^ ± 0.19
	En LP	11.68_A_^a^ ± 0.54	11.57_A_^a^ ± 0.38	11.37_A_^a^ ± 0.35	11.11_AB_^a^ ± 0.24	11.09_AB_^a^ ± 0.27	11.0_AB_^a^ ± 0.28	10.60_B_^a^ ± 0.51
**pH 4.6-SN/TN**	**C**	8.36_C_^a^ ± 0.65	9.64 _C_^a^ ± 0.95	11.47_BC_^a^ ± 0.81	14.31_B_^a^ ± 2.04	18.78 _A_^a^ ± 1.48	20.72_A_^a^ ± 2.16	21.20_A_^a^ ± 3.07
	Free LPE	8.94_E_^a^ ± 0.49	9.49 _E_^a^ ± 0.61	12.03_D_^a^ ± 1.33	14.91 _C_^a^ ± 1.71	20.07 _B_^a^ ± 1.26	22.68_A_^a^ ± 2.35	24.51_A_^a^ ± 1.02
	Co-en LPE	8.40_D_^a^ ± 0.57	8.93 _D_^a^ ± 0.76	10.29_D_^a^ ± 1.21	13.21 _C_^a^ ± 1.46	17.57 _B_^a^ ± 1.94	21.35_A_^a^ ± 1.11	23.06_A_^a^ ± 0.93
	En LP	8.67_D_^a^ ± 0.76	10.19_D_^a^ ± 1.34	10.09 _D_^a^ ± 2.24	13.34 _C_^a^ ± 2.50	18.61 _B_^a^ ± 1.88	20.41_AB_^a^ ± 1.05	22.29_A_^a^ ± 1.76
**TCA/TN**	**C**	1.85_D_^c^ ± 0.15	1.71 _D_^b^ ± 0.43	1.87 _D_^c^ ± 0.17	2.34 _C_^c^ ± 0.43	3.16 _B_^c^ ± 0.19	4.89_A_^c^ ± 0.14	5.03 _A_^c^ ± 0.08
	Free LPE	2.86_E_^a^ ± 0.22	3.67_D_^a^ ± 0.58	4.39 _D_^a^ ± 0.44	5.68 _C_^a^ ± 0.57	7.45 _B_^a^ ± 0.44	8.69_A_^a^ ± 0.27	8.56 _A_^a^ ± 0.19
	Co-en LPE	2.23_E_^b^ ± 0.17	2.25 _E_^b^ ± 0.46	3.27 _D_^b^ ± 0.55	4.21 _C_^b^ ± 0.59	6.29 _B_^b^ ± 0.46	6.17_B_^b^ ± 0.62	7.23_A_^b^ ± 0.18
	En LP	2.29_D_^b^ ± 0.11	2.78 _D_^ab^ ± 0.68	2.96 _D_^b^ ± 0.48	4.07 _C_^b^ ± 0.24	6.55 _B_^b^ ± 0.35	5.92_B_^b^ ± 0.73	7.36_A_^b^ ± 0.12

† a, b, c–For each day of storage, different lowercase superscripts in a column signify significant differences (P ≤ 0.05) between different treatments.

‡ A, B, C–For each treatment, different capital superscripts in a row signify significant differences (*P* ≤ 0.05) between different days of storage.

⁎ The values represent the means of three samples (*n* = 3).

Rashidinejad et al. (2013) noted that the AA of cheese extracts relates to the rate of proteolysis and the period of aging, and accordingly, the production of water-soluble peptides and free amino acids is an important factor for alterations in the antioxidant capacity of the cheeses. Synbiotic cheese Co-enLPE presents the highest values of TPC and AA among cheeses because of the SMSE adding, known as silymarin with potent antioxidant activity (Mudge et al., 2015). On the other hand, the encapsulation process protected the SMSE from chemical instability (oxidation or degradation, etc) during the cheese storage period. The existence of SMSE caused a marked increase of the TPC and AA in the fortified synbiotic cheeses with free and co-encapsulated SMSE relative to the extract-free samples (C and EnLP). Similarly, Sardiñas-Valdés et al. (2021) reported that the supplementation of manchego-style cheeses with nano-emulsified curcumin increased the TPC and AA significantly. In the cheese FreeLPE, the TPC decreased remarkably from 284.27 ± 1.12 to 214.58 ± 3.14 mg Gallic acid equivalents (GAE) per 100 g of cheese (about 24% reduction) at 20 days of aging and accordingly, its AA sharply decreased afterward increased gradually. The fast decrease at 20 days could be due to the decomposition of SMSE, oxidative damage, and or the diffusion of it into the serum phase or biosorption of the SMSE on the probiotic surface. Another reason could be due to the different interactions between milk proteins and free SMSE. Although the cheese FreeLPE had higher TPC levels than the cheese EnLP, no significant difference was seen in terms of AA after 30 days. This may probably be due to the structure of produced bio-compounds since the sequence of bio-peptides is substantial in having their antioxidant specification. Peptides with a Pro-His-His sequence present the highest AA (Moghaddas Kia et al., 2018). On the other hand, it is known that, the Folin-Ciocalteu method can detect also reducing substances and non-antioxidant phenolic compounds in the sample (Brand-Williams et al., 1995).

Cheese EnLP presented a higher TPC and AA than the control during the aging time. This probably could be related to the proteolysis activity of the incorporated probiotic cells on protein breakdown and liberation of small peptides, amino acids, amines, urea, and ammonia (Chen et al., 2019). This confirms the results obtained from secondary proteolysis (TCA-SN; Table 2). As the obtained values for TCA-SN/TN in cheese C were 1.85%, 1.71%, 1.87%, 2.34%, 3.17%, 4.89 and 5.03% while in the cheeses containing encapsulated L. *plantarum*, the mean values were 2.29%, 2.78%, 2.96%, 4.07%, 6.55%, 5.92%, and 7.36%. Chen et al. (2019) noted that *Lactobacillus rhamnosus* in cheeses incorporated with them significantly could increase the proteolytic activity (*P* < 0.05) by producing small polypeptides during the ripening time. Moghaddas Kia et al. (2018) measured AA of Feta cheese containing *Lactobacillus paracasei* and found 33% scavenging activity at 45 days storage, while sample Co-enLPE was able to reach 31% at 60 days despite of SMSE adding. During ripening, the hydrolysis of fat results in increased free radical concentrations especially in soft cheeses. It should be noted that the antioxidant activity obtained in this study may not be real numbers due to the possibility of oxidative damage during cheese ripening. So it is possible that part of the antioxidant activity of SMSE is participated to remove free radicals resulting from the oxidation of lipids.

### 3.5 Chemical and biochemical composition of the enriched cheeses with free, encapsulated, and co-encapsulated cells

Chemical and biochemical properties such as moisture, syneresis percentage, acidity, pH values, fat percentage, and protein content as well as, pH 4.6-SN and TCA-SN percentage for Feta cheese samples during refrigerated storage are shown in Table 3. No significant differences *p* ≤ 0.05 were observed among the cheese samples in terms of the moisture, protein, and fat indicating incorporation of probiotic cells or SMSE has no direct effect on cheese composition. These results confirms the reports of Ong et al. (2006), Ribeiro et al. (2016). According to Table 3, all cheese samples showed a significant decrease in moisture (*P* ≤ 0.05; approximately 4 to 6%) and a simultaneous increase in syneresis percentage. The significant decrease in moisture content can be due to osmosis and syneresis process during ripening.

**Table 3:** The texture profile analysis (TPA) of the different treatments of whey-less Feta-type cheese during storage at 4̊ C.

	Cheese samples	Storage times ^†^_‡_ (days)⁎						
		**1**	10	20	30	**40**	**50**	**60**
**Hardness (N)**	C⁎	1.91_D_^a^ ± 0.13	2.24_CD_^a^ ± 0.40	2.55_C_^b^ ± 0.21	3.32_B_^a^ ± 0.14	3.61_AB_^a^ ± 0.27	3.95_A_^a^ ± 0.19	3.80_A_^a^ ± 0.26
	Free LPE	2.24_C_^a^ ± 0.23	2.77_AB_^a^ ± 0.14	2.96_A_^a^ ± 0.17	2.61_B_^b^ ± 0.09	2.40_BC_^c^ ± 0.06	2.43_BC_^c^ ± 0.14	2.58_B_^c^ ± 0.22
	Co-en LPE	1.97_C_^a^ ± 0.46	2.19_C_^a^ ± 0.38	2.03_C_^c^ ± 0.25	2.46_BC_^b^ ± 0.11	2.89_AB_^b^ ± 0.24	2.81_AB_^b^ ± 0.18	3.03_A_^bc^ ± 0.29
	En LP	1.81_C_^a^ ± 0.57	2.15_BC_^a^ ± 0.12	2.33_B_^bc^ ± 0.11	2.28_BC_^b^ ± 0.27	2.81_A_^b^ ± 0.15	2.99_A_^b^ ± 0.14	3.09_A_^b^ ± 0.17
**Gumminess (N)**	C	1.38_D_^a^ ± 0.11	1.64 _C_^a^ ± 0.31	1.76 _C_^b^ ± 0.17	2.33 _B_^a^ ± 0.10	2.64 _AB_^a^ ± 0.16	2.85_A_^a^ ± 0.18	2.71 _A_^a^ ± 0.23
	Free LPE	1.65_B_^a^ ± 0.16	1.96 _AB_^a^ ± 0.11	2.13 _A_^a^ ± 0.14	1.44 _C_^c^ ± 0.07	1.35_C_^c^ ± 0.05	1.31_C_^c^ ± 0.12	1.35 _C_^c^ ± 0.19
	Co-en LPE	1.43_C_^a^ ± 0.31	1.62 _C_^a^ ± 0.25	1.45 _C_^c^ ± 0.13	1.83_BC_^b^ ± 0.09	2.19_AB_^b^ ± 0.16	2.13_AB_^b^ ± 0.14	2.27_A_^b^ ± 0.17
	En LP	1.36_C_^a^ ± 0.38	1.61 _B_^a^ ± 0.10	1.72 _BC_^b^ ± 0.09	1.73 _BC_^b^ ± 0.21	2.13 _A_^b^ ± 0.12	2.26_A_^b^ ± 0.11	2.35_A_^ab^ ± 0.14
**Chewiness**	C	1.30_D_^a^ ± 0.09	1.61 _C_^a^ ± 0.18	1.67 _C_^ab^ ± 0.13	2.23 _B_^a^ ± 0.07	2.56 _AB_^a^ ± 0.09	2.73_A_^a^ ± 0.15	2.57_AB_^a^ ± 0.19
	Free LPE	1.57 _C_^a^ ± 0.11	1.84 _B_^a^ ± 0.10	2.07 _A_^a^ ± 0.12	1.31 _D_^c^ ± 0.05	1.22 _D_^c^ ± 0.03	1.15_E_^c^ ± 0.09	1.11_E_^c^ ± 0.15
	Co-en LPE	1.36_C_^a^ ± 0.24	1.52 _BC_^a^ ± 0.17	1.39 _C_^b^ ± 0.11	1.78_B_^b^ ± 0.06	2.08_AB_^b^ ± 0.14	2.04_AB_^b^ ± 0.11	2.15_A_^b^ ± 0.12
	En LP	1.31_D_^a^ ± 0.29	1.57_C_^a^ ± 0.08	1.62_BC_^ab^ ± 0.06	1.68_BC_^b^ ± 0.17	2.04 _B_^b^ ± 0.09	2.13_A_^b^ ± 0.10	2.28_A_^b^ ± 0.11
**Springiness**	C	0.92_A_^a^ ± 0.02	0.93_A_^a^ ± 0.04	0.91_A_^a^ ± 0.07	0.92_A_^a^ ± 0.10	0.93_A_^a^ ± 0.02	0.92_A_^a^ ± 0.05	0.92_A_^a^ ± 0.03
	Free LPE	0.91_A_^a^ ± 0.038	0.90_A_^a^ ± 0.01	0.93_A_^a^ ± 0.04	0.85_AB_^a^ ± 0.07	0.86_AB_^a^ ± 0.05	0.81_B_^a^ ± 0.02	0.80_B_^b^ ± 0.06
	Co-en LPE	0.91_A_^a^ ± 0.04	0.92_A_^a^ ± 0.07	0.90_A_^a^ ± 0.08	0.94_A_^a^ ± 0.01	0.90_A_^a^ ± 0.02	0.92_A_^a^ ± 0.09	0.91_A_^a^ ± 0.01
	En LP	0.93_A_^a^ ± 0.09	0.93_A_^a^ ± 0.05	0.90_A_^a^ ± 0.04	0.92_A_^a^ ± 0.02	0.93_A_^a^ ± 0.04	0.90_A_^a^ ± 0.06	0.92_A_^a^ ± 0.08
**Cohesiveness**	C	0.72_A_^a^ ± 0.02	0.73 _A_^a^ ± 0.04	0.69 _A_^a^ ± 0.07	0.70_A_^a^ ± 0.08	0.73 _A_^a^ ± 0.06	0.72_A_^a^ ± 0.11	0.71_A_^a^ ± 0.04
	Free LPE	0.73_A_^a^ ± 0.01	0.70_A_^a^ ± 0.03	0.71_A_^a^ ± 0.06	0.54_B_^a^ ± 0.09	0.55_B_^b^ ± 0.01	0.53_B_^b^ ± 0.04	0.51_B_^b^ ± 0.02
	Co-en LPE	0.71_A_^a^ ± 0.10	0.72_A_^a^ ± 0.12	0.70_A_^a^ ± 0.11	0.73_A_^a^ ± 0.07	0.72_A_^a^ ± 0.09	0.73_A_^a^ ± 0.05	0.73_A_^a^ ± 0.01
	En LP	0.72_A_^a^ ± 0.01	0.71_A_^a^ ± 0.02	0.72_A_^a^ ± 0.03	0.73_A_^a^ ± 0.09	0.72_A_^a^ ± 0.08	0.71_A_^a^ ± 0.03	0.72_A_^a^ ± 0.04
**Adhesiveness (N s)**	C	−0.24_A_^a^ ± 0.54	−0.23_A_^a^ ± 0.36	−0.20_A_^a^ ± 0.97	−0.23_A_^a^ ± 0.57	−0.22_A_^a^ ± 0.48	−0.24_A_^a^ ± 0.33	−0.25_A_^a^ ± 0.51
	Free LPE	−0.20_A_^a^ ± 0.28	−0.22_A_^a^ ± 0.27	−0.20_A_^a^ ± 0.39	−0.25_A_^a^ ± 0.18	−0.23_A_^a^ ± 0.34	−0.23_A_^a^ ± 0.65	−0.21 _A_^a^ ± 0.44
	Co-en LPE	−0.21_A_^a^ ± 0.19	−0.21_A_^a^ ± 0.37	−0.22_A_^a^ ± 0.42	−0.23_A_^a^ ± 0.51	−0.23_A_^a^ ± 0.16	−0.25_A_^a^ ± 0.32	−0.23 _A_^a^ ± 0.29
	En LP	0.22_A_^a^ ± 0.26	−0.24_A_^a^ ± 0.38	−0.23_A_^a^ ± 0.41	−0.24_A_^a^ ± 0.45	−0.23_A_^a^ ± 0.16	−0.25_A_^a^ ± 0.12	−0.21_A_^a^ ± 0.14

† a, b, c–For each day of storage, different lowercase superscripts in a column signify significant differences (P ≤ 0.05) between different treatments.

‡ A, B, C–For each treatment, different capital superscripts in a row signify significant differences (P ≤ 0.05) between different days of storage.

⁎ See Table 1 for description of treatments C, Free LPE, Co-en LPE and En LPE.

All the cheeses indicated increased syneresis (*p* < 0.05) during storage. Irregularly some days of ripening the whey was anew absorbed into the cheese, this occurs through increased proteolysis, continued by an increase in hydrophobic inners in curd thus water absorption increased. The syneresis rate is reversely attributed to pH and thus directly affected by acidity (de Oliveira et al., 2012; Souza & Saad, 2009). In this study, this relation was obtained for cheeses so that cheese FreeLPE presented the lowest pH (3.71) and the highest acidity (1.58%) at the end of storage (Table 2), illustrating the highest percentage of syneresis (10.77%) at the same time. This indicated that free L. *plantarum* cells had a high acidifying activity and were capable of promoting lactose fermentation and lactic acid production. All of the cheeses showed a significant decrease (*p* ≤ 0.05) in pH during the first 40 days, after which a slight increase was observed concomitant to the increase and reduction in titratable acidity (Table 2). Increased pH at the end of storage could be due to the usage of lactic acid, the creation of nonacidic disintegration products, and the release of alkaline products of protein disintegration. In contradiction of the pH value and moisture content, fat levels of cheese samples increased (relatively little; *P* ≤ 0.05) during storage while there was no significant difference in cheese Co-enLPE. The increase in fat levels could be due to the reduction of moisture content during storage. Regarding protein content, the cheese samples indicated a significant reduction trend during storage (P ≤ 0.05). These alterations were statistically considerable (P ≤ 0.05). A decreasing trend in protein could be due to the hydrolysis of protein to short-chain peptides and amino acids and the production of water-soluble nitrogen compounds and the diffusion of these compounds during syneresis. The proteolysis pattern in the cheese samples by determination of pH 4.6-SN and TCA-SN during 60 days of storage time at 4 °C is evaluated. pH 4.6-SN /TN index in all cheeses increased continually during the storage time. There were no significant differences p ≤ 0.05 among probiotic and control cheeses throughout the ripening time. As the ratio of pH 4.6-SN to TN is known as primary proteolysis and is produced by the coagulant (Ong et al., 2006) obtained result was expected. On the contrary, the level of TCA-SN/TN (secondary proteolysis) was significantly different (*p* < 0.05) among cheese samples and increased significantly in all cheese samples during the ripening period. Proteinases of starter and non-starter bacteria are mostly agents of the production of TCA-SN (de Oliveira et al., 2012). The highest and lowest amount of TCA-SN was found in synbiotic cheese FreeLPE and cheese C, respectively, indicating the free L. *plantarum* in special has a more active peptidolytic ability than starter culture and its encapsulated form. This enzymatic system considerably affected the production of medium or small-sized peptides and free amino acids throughout storage time, even at 1 day of ripening, compared with a starter culture. These results accord with the trend found by TPC and AA analyses suggesting the lowest TPC and AA in cheese control (Table 1). Although, the increase in AA, which was in line with the increase of TCA-SN in cheeses during ripening, was not observed in cheese FreeLPE. This result shows the mutual effect of starter culture and probiotic especially in free form and the predominance of free probiotics for donating peptidolytic ability. Kasimoǧlu et al. (2004) reported that released bacteriocins from the L. *plantarum* (plantasin) could alter starter bacteria growth (Kasimoǧlu et al., 2004). The decreased AA in cheese FreeLPE despite having the highest amount of TCA-SN during ripening indicates that the antioxidant peptides were not resistant to further proteolysis and have been changed and converted to new peptides without antioxidant properties (Gupta et al., 2009). This shows that the free or micro-encapsulated state of the probiotic has an important effect on the performance and activity of the starter culture, so that this effect is greater in the free form which following mutual effect. In terms of cell survivability, free *L. plantarum* viability decreased by approximately 3 log at the end of storage, while encapsulated or co-encapsulated form reduced by only about 1 log (Fig. 3). Unexpectedly differences in probiotic viability do not demonstrate the obtained differences in secondary proteolysis (peptidolytic activity). Also, differences in TCA-SN level were obtained even at 1, 10, and 20 days, when no significant difference was found in the viability of the free, encapsulated, and co-encapsulated L. *plantarum* (*p* ≤ 0.05; Fig. 3). Similarly, Gardiner et al. (1998) reported that adjunct *lactobacillus* at high levels of survivability or low levels (Regardless of the number of cells), increased the production of the free amino acid in Cheddar cheese. This study showed enzymatic ability is more dependent on the free or encapsulated form of the strain than the number of cells.

Also, one should not ignore the possibility of free bacterial lysis which results in the liberation of intracellular peptidases inside the curd mass. Hannon et al. (2006) reported that the extent of lysis of *Lb. helveticus* in UF cheese was dependent on the applied strains and species and was started from the onset of storage. Autolysis of starter and nonstarter lactic acid bacteria (NSLAB) resulting in released peptidases subsequently causes cheese proteolysis. Beforehand, bacterial lysis of lactic acid bacteria in cheeses is reported as an approach to the acceleration of biochemical processes implicated in proteolysis reactions (El Soda, 1993), thus this is a plausible comment for the significant increase of TCA/TN by the free cells compared with the encapsulated or co-encapsulated cells since the decreased viability of free cells could correspond to increased lysis of the cell. This suggested that the released enzymes were more stable in the cheese mass than free cells. On the other hand, the level of soluble nitrogen in TCA did not present significant differences (*p* > 0.05) between probiotic cheese EnLP and synbiotic cheese Co-enLPE during ripening indicating SMSE added to synbiotic cheese Co-enLPE did not affect the formation of peptides and other nitrogenous compounds such as amino acids and amines. These results agreed with observations noted by Sardiñas-Valdés et al. (2021) to Manchego-style cheese produced with nano-emulsified curcumin (NEC). Those observations present that the incorporation of the NEC into the hair sheep milk did not change the primary and secondary proteolysis of cheese.

### 3.6 Texture profile analysis

**Fig. 4:**
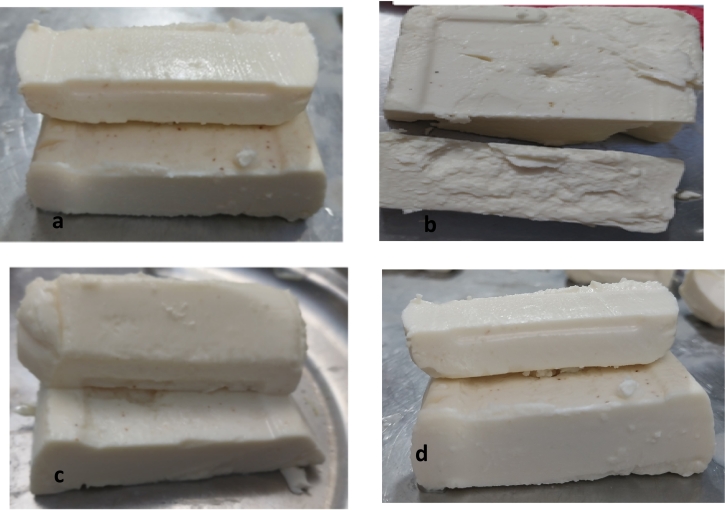
Visual appearance of the synbiotic Feta-type cheeses (a) C, (b) Free LPE, (c) Co-en LPE and (d) En LPE.

The texture is one of the most important quality characteristics in the sensory acceptability of cheese. Fig. 4 indicates the visual appearance of the cheeses and Table 3 the various rheological parameters acquired quantitatively for cheeses C, FreeLPE, EnLP, and Co-enLPE during ripening. Hardness is the most significant texture feature because less stiffness cheeses with fragile texture are ordinarily refused by consumers.

Hardness, gumminess, and chewiness indicated significant changes among of cheeses during ripening, while cohesiveness, springiness, and adhesiveness remained steady with the exception of cheese FreeLPE, particularly after 20 days. Encapsulation type and storage time influenced statistically in the hardness of cheese samples (*P* ≤ 0.05). Cheeses showed a non-significant difference in hardness between 1 and 10 days of storage, followed by a significant difference afterward as the highest and lowest hardness were obtained for the cheeses C and cheeses FreeLPE, respectively. Hardness in all of the cheese samples increased continuously during storage while cheese FreeLPE showed a significant increase up to 20 days followed by a significant decrease afterward. Continuous increase in hardness of the cheeses C, EnLP, and Co-enLPE could be related to a decrease in moisture and an increase in syneresis created by concomitant acidification of L. *plantarum* and starter. Moisture behaves as a plasticizer in the protein matrix, thus leading to less elasticity and more fracturability upon compression. Moisture loss increases the contact surface between the chains of casein and leads to increased electrostatic interactions and hydrogen bonds (Salazar-Montoya et al., 2018). In terms of synersis effect, it can be explained as follows. By increasing syneresis during ripening, the type and concentration of compounds that were transferred to the serum phase as a result of mild proteolysis (Table 2), could not weaken the network structure of curd leading to the formation of a hard curd with the exit of excess moisture in cheeses C, EnLP, and Co-enLPE. While in cheese FreeLPE, transferred water-soluble peptides in the serum phase could not participate in the network structure of curd leading to the formation of a softer curd. This result confirms that the intensity of proteolysis has a significant effect on the transfer of various compounds to the serum phase, which leads to the hardness or softness of the cheese. The color of the opac white to the bright white of the serum phase was visually observed during the experiment.

In the cheese Free LPE initial increase in hardness could be probably due to moisture loss, while the subsequent significant decrease was influenced by the higher acidification rate and greater proteolytic activity (higher values of titratable acidity and higher TCA-SN concentration; Table 2) of the free L. *plantarum*. Comparing texture values and pH of the cheeses (Table 3, Table 2) shows a direct dependency between hardness and pH. At low pH values due to the solubilization of colloidal calcium phosphate crosslinks, demineralization of casein micelles occurs resulting in a reduction in firmness (Souza & Saad, 2009). Also at low pHs casein micelles become strand form and break at their yield point while at higher pHs, protein agglomerations have bigger spherical forms and flow freely under applied force (El-Salam et al., 1999). It seems that the fortifying effect of moisture loss or increased syneresis during ripening prevails on the attenuating effect of increased proteolysis in the cheeses C, Co-enLPE and EnLP. Although TCA soluble N concentration in cheese Co-enLPE and cheese EnLP was higher than in cheese C, it seems sensible considering that proteolysis intensity not be as high as in cheese FreeLPE, which caused a softer curd as a result of the release of nitrogen compounds to serum phase. This indicates that despite increased secondary proteolysis is still exist some internal bonds and electrostatic interactions which prevent the transfer of soluble nitrogenous compounds to the serum phase in synbiotic cheese Co-enLPE and probiotic cheese EnLP.

In the same way, a decrease of cheese firmness by increasing proteolysis rate has been presented in different studies such as Moghaddas Kia et al. (2018) in probiotic UF Feta cheese, Souza and Saad (2009) in probiotic Minas cheese. Moghaddas Kia et al. (2018) noted that water-soluble broken peptides obtained from proteolysis transfer the serum phase and cannot participate in the network structure of curd leading to the formation of a softer curd with lower amounts in hardness. In line with changes in hardness, gumminess, and the amount of energy needed for chewing the cheeses were altered similarly. In cheeses C, Co-enLPE, and EnLP the chewiness increased over time and had a positive correlation with hardness suggesting more bites are required to break down the cheese structure.

There were no significant differences (*P* ≤ 0.05) in terms of springiness, cohesiveness, and adhesiveness among cheeses with the exception of synbiotic cheese FreeLPE and all cheeses maintained constant levels during ripening. Exceptionally cheese FreeLPE revealed a lower amount of cohesiveness (significant decrease between 40 and 60 days; *P* < 0.05) and springiness (significant decrease only at 60 days; P < 0.05) among cheese samples after 20 days which was probably due to soft texture created by excessive proteolysis activity. Similarly, a negative correlation between springiness and the proteolysis progress was reported by Salazar-Montoya et al. (2018) which attributed the loss of elasticity in Manchego-type cheese during storage to protein breakdown.

Structural damages created by compression can increase in the softer sample (cheese FreeLPE) leading to a lower cohesiveness and springiness. The Relationship between chemical composition and cohesiveness has allocated opposite and similar results in the studies. Some studies reported a negative correlation between cohesiveness and hardness as well as cohesiveness and dry mass content (Delgado et al., 2011; Eroglu et al., 2016) during cheese storage. Furthermore, reported a negative correlation between gumminess/chewiness and hardness in 4 types Kashar cheese during storage (Eroglu et al., 2016). Similarly, another study reported an increase in syneresis (from 2 to 19%), hardness, gumminess, and chewiness concomitant with the approximately constant value of cohesiveness of Minas fresh cheese during storage (Souza & Saad, 2009). Some studies found a positive correlation between cohesiveness and the dry matter content (Koca & Metin, 2004) and between cohesiveness and hardness (Moghaddas Kia et al., 2018) during storage in cheese. According to the results of the previous and present studies, establishment of a correct relation between the physicochemical properties and cohesiveness of the cheese samples is impossible because cheese is composed of different compounds (fat, protein, ash) with a very complex material. Furthermore, glycolysis, proteolysis, and lipolysis occurring during storage are also effective agents on the intensity of the bonds composing the cheese structure. Determining the cause-and-effect relation is impossible because the strength between the bonds that compose the structure of the cheese is affected by different agents concurrently.

## 4 Conclusion

In this study for the first time, the effect of the addition of co-encapsulated L. *plantarum* (MT, ZH593) and SMSE on cheese physicochemical composition, proteolytic pattern and texture parameters as well as cell survivability was evaluated. This research demonstrates that co-encapsulated SMSE and L. *plantarum* applied in cheese is promising due to the embedding two bioactive compounds, which can be liberated and show possible synergistic effects. Furthermore, the addition of co-encapsulated SMSE, however, had no significant effect on the physicochemical parameters of cheeses, but significantly increased antioxidant activity and the survivability of probiotic population in the cheese and sailin solution during storage, indicating a potential prebiotic capacity of SMSE as a new non-carbohydrate prebiotic. According to the obtained results, encapsulation type had no significant influence on moisture content, protein, and fat level of cheeses (*p* ≤ 0.05) while cell survivability, stability of polyphenols, antioxidant activity, titrable acidity, TCA-SN, and pH were influenced by both encapsulation type and ripening time. The results indicated cheeses incorporated with free cells despite having the highest TCA-SN concentration (secondary proteolysis indicator) demonstrated the lowest viability among cheeses during ripening, indicating probably has occurred bacterial lysis and liberation of intracellular peptidases in the curd matrix. Meanwhile, this investigation did not study cell lysis, thus supplementary study may be required on this issue. In this study, we found that the native probiotic L. *plantarum* in free form has strong proteolytic activity and therefore we recommend its use in hard cheeses or in soft cheese without using starter.

It can be suggested that simultaneous usage of the probiotic bacteria and SMSE in WPI-coated microbeads instead of the individual usage of them in the production of functional and synbiotic foods as well as food supplements.

Since this study is the first research fully focused effect of the addition of co-encapsulated *L. plantarum* (MT, ZH593) and SMSE in cheese, it was not investigated oxidative spoilage/lipolysis and microbial spoilage during ripening. Thus, it is suggested that application of the co-encapsulated SMSE and probiotic as an alternative bio-preservation in order to increase the shelf life of the cheese according to synergistic antimicrobial and antioxidant activities between SMSE and probiotic be investigated.

## CRediT authorship contribution statement

**Maryam Bakhtiyari:** Writing – original draft, Validation, Resources, Investigation. **Zohreh Hamidi-Esfahani:** Writing – review & editing, Supervision, Software, Conceptualization. **Mohsen Barzegar:** Conceptualization.

## Declaration of competing interest

The authors declare that they have no known competing financial interests or personal relationships that could have appeared to influence the work reported in this paper.

## Data Availability

Data will be made available on request.
